# Evaluation of Functional Marine Protein Hydrolysates as Fish Meal Replacements in Low-Fish-Meal Diets: Effects on Growth Performance, Feed Utilization, and Health Status of Asian Seabass (*Lates calcarifer*)

**DOI:** 10.3390/ani15223285

**Published:** 2025-11-13

**Authors:** Dachawat Poonnual, Siriporn Tola, Bundit Yuangsoi

**Affiliations:** Department of Fisheries, Faculty of Agriculture, Khon Kaen University, Khon Kaen 40002, Thailand; dachawat.poonnual@symrise.com (D.P.); siripto@kku.ac.th (S.T.)

**Keywords:** Asian seabass, marine protein hydrolysates, growth performance, feed utilization, health status

## Abstract

An eight-week study was conducted to evaluate the effects of dietary marine protein hydrolysates as fish meal replacements in low-fish-meal diets on the growth performance, feed utilization, and health status of Asian seabass (*Lates calcarifer*). The high-fish-meal (HFM) diet contained 25% fish meal, while the low-fish-meal (LFM) diet replaced 60% of the fish meal with soybean meal. Three experimental diets were formulated by supplementing the LFM diet with 5% tuna hydrolysate (TH), 2% shrimp hydrolysate (SH), and 5% salmon silage (SS), each replacing an equivalent amount of fish meal. These diets were designated as LFM + TH, LFM + SH, and LFM + SS, respectively. The results showed that the LFM + TH diet significantly improved the percentage of weight gain, average daily growth, specific growth rate, protein efficiency ratio, and feed conversion ratio compared to the LFM diet (*p* < 0.05), without negatively affecting feed intake or metabolic markers. Histological analysis revealed improved villus length and goblet cell count in the intestine, indicating better nutrient absorption (*p* < 0.05). However, no significant differences were observed in hematological and immunological parameters, blood plasma metabolic markers, or carcass proximate composition (*p* > 0.05). Furthermore, the LFM + TH diet exhibited superior survival rates under ammonia stress, highlighting its potential to enhance stress tolerance. These findings suggest that marine protein hydrolysates, particularly 5%TH, can serve as a sustainable and efficient alternative to fish meal protein in diets with up to 60% in soybean meal compensation, promoting better growth and survival in Asian seabass.

## Simple Summary

This study tested marine protein hydrolysates from seafood by-products as fish meal replacements in Asian seabass diets. Tuna hydrolysate, when added to soybean meal-based diets, restored growth, feed efficiency, and stress tolerance to levels similar to traditional fish meal diets. Intestinal health improved while other health indicators were unchanged. Tuna hydrolysate shows strong potential as a sustainable fish meal alternative for seabass farming.

## 1. Introduction

Asian seabass (*Lates calcarifer*) is a high-value species and is widely farmed in the Asia Pacific region, particularly in countries like Australia, Indonesia, Malaysia, the Philippines, Taiwan, and Thailand [1,2,3]. As a carnivorous species, it requires a high-protein diet, typically consisting of 40–50% protein [1,2] often sourced from fish meal (FM), which provides an excellent amino acids profile, omega-3, fatty acids, and contributes to dietary palatability [4,5]. However, the increasing cost and demand for FM has led to the exploration of alternative protein sources in aquafeeds.

Despite various studies investigating plant- and animal-based FM substitutes, challenges such as amino acids imbalance, anti-nutritional factors, and reduced palatability remain, impacting growth and utilization in carnivorous diets [6,7,8,9,10,11]. While many plant- and animal-based alternatives to fish meal have been studied, issues like amino acid imbalance and poor palatability limit their use in the feeding of carnivorous fish such as Asian seabass [7,12,13,14,15,16,17].

Marine protein hydrolysates, derived from seafood processing by-products including heads, skin, bones, and viscera [14,18], provide high-quality protein with excellent functional properties, including enhanced palatability, health benefits, and high digestibility. These hydrolysates are rich in bioactive peptides and free amino acids, which not only improve feed palatability but also stimulate feed intake. As a result, they are widely used as feed ingredients in animal feed, offering significant nutritive value [15]. Several studies have highlighted that supplementing 1–6% of protein hydrolysates in low-fish-meal diets improves feed intake, feed utilization, growth performance, and health in aquaculture species such as Asian seabass [7,16], Largemouth Bass (*Micropterus salmoides*) [19,20], Snakehead fish (*Channa striata*) [8,21], *Octopus maya* [22], and Hybrid grouper (*Epinephelus fuscoguttatus* ♀ × *E. lanceolatus* ♂) [23].

Marine protein hydrolysates offer promise due to their bioactive peptides, but direct comparisons among types (tuna, shrimp, and salmon) and their effects on health and stress tolerance in Asian seabass are limited. This highlights the need for a comprehensive evaluation of different marine hydrolysates as fish meal replacements in seabass diets.

Therefore, the present study aims to evaluate the effect of marine protein hydrolysates including tuna hydrolysate (TH), shrimp hydrolysate (SH), and salmon silage (SS) as dietary supplements replacing fish meal on growth performance, feed utilization, nutrient digestibility, health status, histological changes, and stress tolerance in Asian seabass.

## 2. Materials and Methods

### 2.1. Experimental Diets

Five isonitrogenous (48% crude protein), isolipidic (11% crude lipid), and isocaloric (19 MJ kg^−1^) diets were formulated, with all diets being isoenergetic on a gross energy basis. The marine ingredients, tuna hydrolysate (TH), shrimp hydrolysate (SH), and salmon silage (SS), with their chemical compositions, molecular weight distributions, and amino acid profiles are shown in Table 1. TH was derived from tuna viscera via enzymatic hydrolysis, SH from shrimp processing waste (35–45% inedible), and SS from category 3 salmon by-products treated with 2% formic acid and gut enzymes. All marine ingredients were provided by Marine and Feed, SPF Diana Thailand Co., Ltd (Symrise group), Samutsakhon, Thailand. A high-FM diet (HFM) contained 25% fish meal, while a low-FM diet (LFM) replaced 60% of fish meal with soybean meal. Three additional diets were based on LFM, supplemented with 5% TH, 2% SH, or 5% SS (on a %, crude basis), and balanced for essential nutrients.

Experimental diets (Table 2) were prepared by blending powdered ingredients with marine protein hydrolysates, fish oil, and soybean oil, then adding 30% water. The mixture was pelletized using a single-screw extruder (Model EXT15HP3V03, Siam Farm Services Co., Ltd., Lampang, Thailand) with a 2 mm die, dried at 90 °C for 4 h, and stored at −20 °C. Random samples were analyzed for their chemical compositions, peptide profiles, and amino acid profiles (Table 2 and Table 3).

### 2.2. Experimental Animals, Conditions, and Feeding Procedure

The experiment was conducted at the Aqualis APAC testing center, SPF Diana (Thailand) Co., Ltd., Samutsakhon, Thailand, using juvenile Asian seabass with body weights of 1–2 g. The fish were acclimatized to freshwater for two weeks and fed a commercial diet (PROFEED No. 902, Thai Union Feed Mill PLC, Samutsakhon, Thailand) containing 42% crude protein and 12% crude lipid. The tanks were equipped with a recirculating water system, with maintained optimal water quality parameters including dissolved oxygen (5.42 ± 0.99 mg L^−1^), pH (7.41 ± 0.17), temperature (26.76 ± 1.52 °C), ammonia (0.13 ± 0.06 mg L^−1^), nitrite (0.42 ± 0.10 mg L^−1^), and alkalinity (104.3 ± 21.1 mg L^−1^). A group of 50 fish with a mean initial weight of 2.62 ± 0.01 g fish^−1^ was distributed into 25 500 L plastic PE tanks. Five replicate groups of fish were each fed one of five experimental diets, twice daily (at 08:00 h and 16:00 h) ad libitum for eight weeks, with feeding discontinued once the fish exhibited a reduced feeding response.

### 2.3. Sample Collection

At the end of the 8-week feeding trial, the fish were fasted for 24 h and then individually weighed to determine their final body weights. Prior to sampling, the fish were anesthetized with a 2-phenoxyethanol solution (2 mg L^−1^). Blood samples were collected from the caudal vein of two fish per replicate (a total of 10 fish per treatment) using a 1 mL sterile syringe with a 23-gauge needle containing 1 mL of precooled anticoagulant (heparin injection BP 25,000 IU, Gland Pharma Limited, Hyderabad, India). The blood samples were then transferred to heparinized tubes for hematological and immunological analyses. Blood plasma was separated by centrifugation at 10,000 rpm for 10 min at 4 °C and preserved at −80 °C until use. Following blood collection, the same two fish per replicate tank (10 fish per treatment) were used for sampling intraperitoneal fat, liver, and whole viscera, which were immediately weighed to calculate the intraperitoneal fat index, hepatosomatic index, and viscerosomatic index. Additionally, liver and intestine samples from these same fish were fixed in 10% formalin buffer for histological analysis. Furthermore, two fish per replicate (a total of 10 fish per treatment), along with 10 fish from the initial carcass, were collected and stored at −20 °C for carcass proximate composition analysis.

### 2.4. Analytical Methods

#### 2.4.1. Growth Performance and Feed Utilization

This data was utilized to calculate growth performance, feed utilization, and survival rates using the following equations:

Percent weight gain (%) = 100 × (final body weight (g fish^−1^)—initial body weight (g fish^−1^)/day of feeding (day)

Average daily growth (g day^−1^) = (average final body weight (g fish^−1^)—average initial body weight (g fish^−1^))/day of feeding (day)

Specific growth rate (% day^−1^) = 100 × (in final body weight (g fish^−1^)—in average initial body weight (g fish^−1^)/day of feeding (day)

Feed conversion ratio = dry feed intake (g fish^−1^)/body weight gain (g fish^−1^)

Protein efficiency ratio = 100 × (body weight gain (g fish^−1^)/protein intake (g fish^−1^))

Nitrogen retention (%) = 100 × (nitrogen gain (g fish^−1^)/nitrogen intake (g fish^−1^))

Lipid retention (%) = 100 × (lipid gain (g fish^−1^)/lipid intake (g fish^−1^))

Intraperitoneal fat (%) = 100 × intraperitoneal fat weight (g)/body weight (g fish^−1^)

Hepatosomatic index (%) = 100 × liver weight (g)/body weight (g fish^−1^)

Viscerosomatic index (%) = 100 × viscera weight (g)/body weight (g fish^−1^)

Survival rate (%) = 100 × (initial fish number (fish)—final fish number (fish)/initial fish number (fish)

#### 2.4.2. Hematological Parameter Analysis

The heparinized blood samples were analyzed for hematological parameters. The red blood cell count and white blood cell count were determined using a Neubauer hemocytometer, following the method of Blaxhall and Daisley [25]. The packed cell volume was measured using the standard microhematocrit method and expressed as a percentage, as outlined by England and Walford [26]. Hemoglobin levels were determined using the cyanmethemoglobin method according to Blaxhall and Daisley [27]. The mean corpuscular hemoglobin concentration (MCHC) was calculated based on the method described by Blaxhall and Daisley [25].

#### 2.4.3. Immunological Parameter Analysis

Lysozyme activity was measured using a turbidimetric assay, as described by Parry et al. [27], with slight modifications. Briefly, 10 µL of blood plasma sample was added to 250 µL of 0.2 mg L^−1^ of *Micrococcus lysodeikticus* suspension (ATCC No. 4698, Sigma-Aldric, St. Louis, MO, USA). The reaction was conducted at 25 °C, and absorbance was measured at 540 nm after 0.5 and 5.5 min with a microplate reader (Thermo Fisher Scientific, Vantaa, Finland). One unit of lysozyme activity was defined as the amount of sample which caused a 0.001 min^−1^ reduction in absorbance. The antioxidant enzyme activities, including NBT activity, were assessed by the reduction of nitroblue-tetrazolium (NBT) to blue formazan method adapted from Song and Hsieh [28], superoxide dismutase activity (SOD) was assessed with a 19160 SOD determination kit (Sigma-Aldric, Taufkirchen, Germany), catalase activity (CAT) was measured using the K-CATAL 07/19 (Magazyme, Bray, Ireland), and the clearance efficiency of blood plasma was assessed using a modified method from Kewcharoen and Srisapoome [29].

#### 2.4.4. Blood Plasma Metabolic Markers Analysis

The blood plasma metabolic markers parameters were analyzed, including cholesterol (enzymatic, OXI/PER), triglyceride (enzymatic, OXI/PER), glucose (enzymatic, oxidase), albumin (colorimetric, BGC), total protein (colorimetric, biuret), creatinine (enzymatic, jiffe), aspartate transaminase (Kinetic, IFCC), and alkaline phosphatase (kinetic, IFCC). These analyses were conducted using an automatic biochemistry analyzer (BA400; BioSystems, Barcelona, Spain).

#### 2.4.5. Histological Liver and Intestine Analysis

After being preserved in 10% formalin for 24 h, a histological analysis of the liver and intestines was performed following the methods outlined by Clark [30]. The liver and intestines were carefully dissected using sterilized surgical scissors, and serial histological sections were prepared. The samples were dehydrated by immersion in increasing concentrations of ethanol up to 100%, before being embedded in paraffin. Tissue sections 4–5 µm thick were stained with hematoxylin and eosin (H&E) to assess their general morphology. Images were captured using a digital microscope camera at magnifications of 4×,10×, and 40× (Nikon, NIS-Elements software version 4.50, Tokyo, Japan).

#### 2.4.6. Carcass Composition Analysis

The proximate composition of whole-body fish, including dry matter, crude protein, crude lipid, and crude ash, was analyzed following the methods described by AOAC [31]. The analysis was performed by the Agricultural Development Research Center in northeast Thailand, Faculty of Agriculture, Khon Kaen University: dry matter (AOAC official method 934.01), crude protein (Kjeldahl method, total N × 6.25), crude lipid (AOAC official method 2003.05, Soxhlet extraction), and ash (AOAC official method 942.05).

### 2.5. Digestibility Analysis

#### 2.5.1. *In Vitro* Protein Digestibility of Marine Ingredients and Experimental Diets

The digestive enzymes were extracted from another set of anterior intestine tissues from 10 Asian seabass (approximately 25–30 g in body weight), following the method of Rungruangsak-Torrissen et al. [32]. The anterior intestine tissues were homogenized in 50 mM Tris-HCl buffer, pH 8, containing 200 mM NaCl (1:5 *w*/*v*). The homogenate was centrifuged at 12,000 rpm at 4 °C for 30 min, and supernatant was collected and stored at −20 °C to determine the *in vitro* protein digestibility.

The *in vitro* protein digestibility of the marine ingredients and experimental diets was evaluated using the modified method of Rungruangsak-Torrissen et al. [33] and Rungruangsak-Torrissen [34]. A 30 mg protein sample (dry basis) was incubated with 40 mL of phosphate buffer (50 mM, pH8.2) and 200 µL of chloramphenicol phosphate (0.5%) at 200 rpm, 30 °C, for 24 h, followed by the addition of 500 µL of dialyzed crude enzyme extract. The digested solution was analyzed by mixing 200 µL of the solution with 2 mL phosphate buffer (50 mM, pH 8.2) and 0.1% Trinitrobenzene sulphonic acid (TNBS), then incubating at 60 °C for 1 h. The reaction was stopped with 1 mL HCl (1 M), and protein digestibility was measured spectrophotometrically (Double Beam Spectrophotometer Libra S80, Biochrom Ltd., Cambridge, England) at 420 nm using DL-alanine (Sigma-Aldrich, Taufkirchen, Germany) as a standard.

#### 2.5.2. *In Vivo* Estimation of Apparent Digestibility Coefficients

The *in vivo* digestibility experiment was conducted using a similar protocol as the growth experiment, incorporating 1% chromium oxide (Cr_2_O_3_) in the diet as an inert marker. Fecal samples were collected twice daily from each tank at 10.00 h and 17.00 h by siphoning. The samples were filtered to remove excess water and then stored at −20 °C. Afterward, the fecal samples were dried overnight at 60 °C, ground, and stored at −20 °C for further analysis. Chromium oxide levels in both diets and fecal samples were determined according to the Austreng method [35], and apparent digestibility coefficients (ADCs) were calculated according to the method described in Cho et al. [36].

### 2.6. Ammonia Stress Challenge Test

After the 8-week feeding trial, an ammonia challenge test was conducted to assess the stress tolerance of the experimental animals under elevated ammonia conditions. The 96 h LC50 for total ammonia nitrogen (TAN) had previously been determined to be 49.049 mg L^−1^, corresponding to an un-ionized ammonia nitrogen (NH_3_–N) concentration of 2.01 mg L^−1^ at a pH of 8.02 ± 0.2 and a temperature of 26.9 ± 0.2 °C., as described by Lin and Chen [37]. Before the challenge, fish from each treatment group were pooled and redistributed into three tanks, with 25 fish per tank. Ammonium chloride (NH_4_Cl, >99.5% purity; Ke Huan, Shanghai, China) was diluted to the target concentration. During the experiment, 50% of the water was replaced with water containing the same ammonia concentration, and the fish were starved. Mortality was monitored and recorded every 12 h over a 120 h period. At the end of the challenge, blood samples were collected from six fish (two from each replicate). Blood was individually collected from the caudal vein using a 1 mL sterile syringe fitted with a 23-gauge needle preloaded with 1 mL of precooled heparin (25,000 IU; Gland Pharma Limited, Hyderabad, India). The samples were transferred to heparinized tubes and centrifuged at 10,000 rpm for 10 min at 4 °C to separate plasma. The plasma was stored at −20 °C until cortisol concentrations were determined using the CMAI method (IMMULITE 1000; Siemens Healthineers, Erlangen, Germany).

### 2.7. Statistical Analysis

All data were analyzed using SPSS software version 23. Prior to analysis, the normality of residuals and homogeneity of variances were verified. Percentage data were natural log-transformed (LN) to meet the assumptions of parametric testing. To evaluate the effects of the different marine protein hydrolysate groups on the growth performance, feed utilization, and health indices of Asian seabass, a one-way ANOVA test was performed. When significant differences were detected, Tukey’s HSD post hoc test was applied for multiple comparisons at a significance level of *p* < 0.05. Survival data were analyzed using the Kaplan–Meier method, and survival curves were constructed accordingly. All results are presented as mean ± standard deviation (S.D.).

## 3. Results

### 3.1. Growth Performance and Feed Utilization

In this eight-week study, Asian seabass fed the LFM + TH diet showed significantly better growth (final body weight, body weight gain, average daily growth, and specific growth), feed efficiency, and protein utilization than those fed the LFM, LFM + SH, or LFM + SS diets (*p* < 0.05), and performed similarly to the HFM group (*p* > 0.05). Feed intake was the highest in the HFM, LFM + TH, and LFM + SS groups, while in the LFM + SH group it was the lowest (*p* < 0.05). The best feed conversion ratio was seen in the LFM + TH and HFM diets. Protein efficiency was the highest in the LFM + TH group. No significant differences were found among diets for nitrogen/lipid retention, fat indices, or organ indices. Survival was the lowest in the LFM group (*p* < 0.05) (Table 4).

### 3.2. Hematological and Immunological Parameters

**Figure 1:**
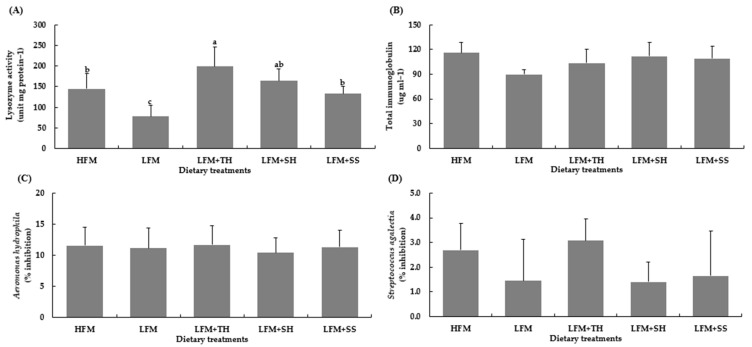
Lysozyme activity (**A**), total immunoglobulin (**B**), and plasma clearance efficiency against *Aeromonas hydrophila* (**C**) and *Streptococcus agalactiae* (**D**) in Asian seabass after 8 weeks of feeding. Values are means ± SD (*n* = 5). Different letters indicate statistical differences (*p* < 0.05) among the treatments. Abbreviations: HFM, high-fish-meal diet; LFM, low-fish-meal diet; LFM + TH, LFM diet + tuna hydrolysate; LFM + SH, LFM diet + shrimp hydrolysate; and LFM + SS, LFM diet + salmon silage.

The hematological and immunological parameters, including red blood cell count (RBC) and white blood cell count, hematocrit, hemoglobin, mean cell hemoglobin concentration (MCHC), nitro tetrazolium activity (NBT), superoxide dismutase activity (SOD), and catalase activity (CAT), did not show any significant differences compared to both the HFM and LFM diets (*p* > 0.05) (Table 5). However, the lysozyme activity in fish fed the LFM + TH diet was significantly higher than in the other diets (*p* < 0.05), although no significant difference was observed compared to the LFM + SH diet (*p* > 0.05). There were no significant differences in lysozyme activity between the fish fed the HFM, LFM + SH, and LFM + SS diets (*p* > 0.05), while the LFM diet exhibited significantly lower activity than all other diets (*p* < 0.05) (Figure 1A). Total immunoglobulin (total Ig) levels did not differ significantly across all the dietary treatments, ranging from 89.61 to 115.61 µg mL^−1^ (*p* > 0.05) (Figure 1B). The plasma clearance efficiency, which assessed the inhibitory effect against *Aeromonas hydrophila* (10.40% to 11.68%) and *Streptococcus agalactiae* (1.41% to 3.07%), showed no significant differences across all dietary treatments (*p* > 0.05) (Figure 1C,D).

### 3.3. Blood Plasma Metabolic Markers

The analysis of blood plasma metabolic markers in Asian seabass, including cholesterol, triglyceride, glucose, albumin, total protein, creatinine, aspartate transaminase, and alkaline phosphatase, revealed no significant differences between the dietary treatments (*p* > 0.05) (Table 6). These results indicated that the dietary treatments did not significantly influence the metabolic parameters assessed in Asian seabass.

### 3.4. Histological Analysis of Liver and Intestine

**Figure 2:**
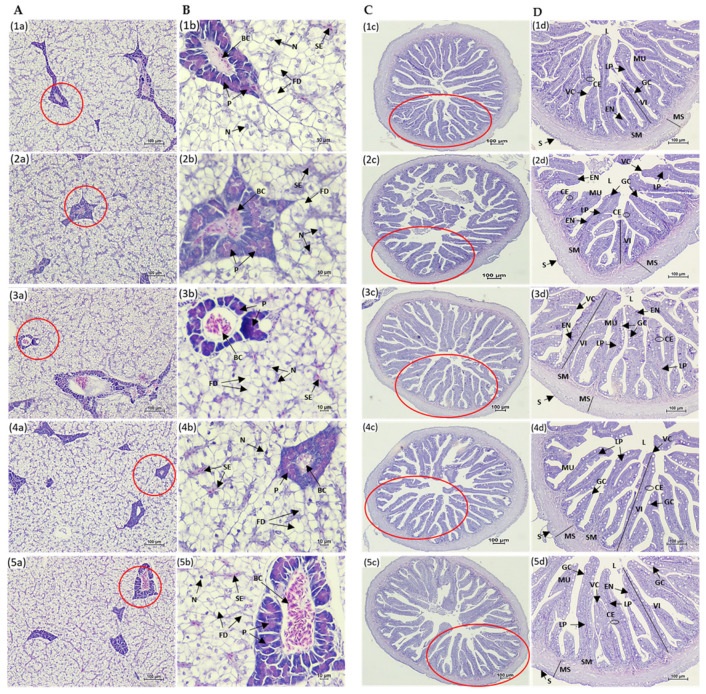
Histological sections of the liver (**A**,**B**) and distal intestine (**C**,**D**) of Asian seabass after 8 weeks of feeding. Panels: (1a–d) HFM; (2a–d) LFM; (3a–d) LFM + TH; (4a–d) LFM + SH; and (5a–d) LFM + SS. Images at 4× magnification (**A**,**C**) and 40× magnification (**B**,**D**). Panels (**B**,**D**) were taken from the regions indicated by the red circles in panels (**A**,**C**), respectively. Abbreviations: CE, columnar epithelium; EN, enterocytes; GC, goblet cells; L, lumen; LP, lamina propria; MU, mucosa; MS, muscularis; VC, villous core containing blood and lymph capillaries; VI, villi; S, serosa; SM, submucosa; N, nuclei; FD, fat deposits; SE, sinusoid erythrocytes; P, pancreatic cells; and BC, blood congestion.

**Figure 3:**
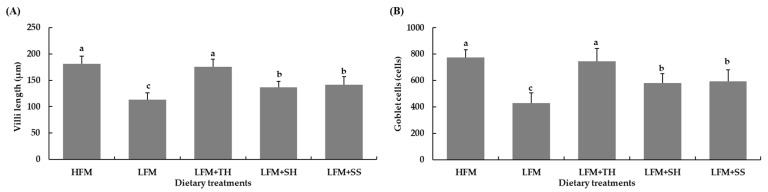
Villi length (**A**) and goblet cell counts (**B**) in the distal intestine of Asian seabass after 8 weeks of feeding. Values are presented as means ± SD (*n* = 5). Different letters indicate statistical differences (*p* < 0.05) among the treatments. Abbreviations: HFM, high-fish-meal diet; LFM, low-fish-meal diet; LFM + TH, LFM diet + tuna hydrolysate; LFM + SH, LFM diet + shrimp hydrolysate; and LFM + SS, LFM diet + salmon silage.

The histological examination of the liver and distal intestine of Asian seabass fed the experimental diets for 8 weeks revealed distinct differences (Figure 2A–D). Fish from all treatment groups exhibited normal liver parenchyma structures, with hepatocytes cords separated by sinusoids containing erythrocytes. The hepatocytes were large, with centrally located nuclei, and displayed prominent nucleoli (Figure 2A,B). Fish fed the HFM diet exhibited normal distal intestine structure, with elongated villi, absorptive vacuoles in the enterocytes, and well-defined villous core containing blood and lymph capillaries (Figure 2(1c,1d)). In contrast, fish fed the LFM diet had a shorter villus in the distal intestine (Figure 2(2c,2d)). Fish fed the LFM + TH diet had a distal intestine structure similar to the HFM group (Figure 2(2c,2d)), with notable increases in villi length and goblet cells significantly higher than those fed the LFM, LFM + SH, and LFM + SS diets (*p* < 0.05), though no significant difference was found compared to the HFM diet group (*p* > 0.05) (Figure 3A). Fish fed the LFM + SH and LFM + SS diets displayed similar villi length and goblet cell counts, with no significant difference between these groups (*p* > 0.05) (Figure 2(4c,4d,5c) and Figure 3A,B). The LFM diet group showed significantly shorter villi and fewer goblet cells compared to other diets (*p* < 0.05) (Figure 2(2c,2d) and Figure 3A,B).

### 3.5. Carcass Proximate Composition

The carcass proximate composition of whole-body fish which were fed the experimental diets for 8 weeks is shown in Table 7. Carcass dry matter, protein, lipid, and ash were not affected by the dietary treatments (*p* > 0.05).

### 3.6. Nutrient Digestibility

#### 3.6.1. *In Vitro* Protein Digestibility of Marine Ingredients and Experimental Diets

**Figure 4:**
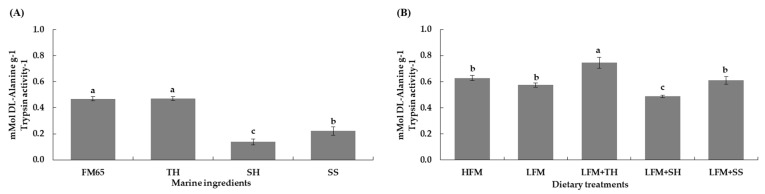
*In vitro* protein digestibility of marine ingredients (**A**) and experimental diets (**B**). Values are presented as means ± SD (*n* = 5). Different letters indicate statistical differences (*p* < 0.05) among the treatments. Abbreviations: FM65, fish meal 65% crude protein; TH, tuna hydrolysate; SH, shrimp hydrolysate; SS, salmon silage; HFM, high-fish-meal diet; LFM, low-fish-meal diet; LFM + TH, LFM diet + tuna hydrolysate; LFM + SH, LFM diet + shrimp hydrolysate; and LFM + SS, LFM diet + salmon silage.

The protein digestibility of marine hydrolysates in the fish meal (65% crude protein) and TH groups (both 0.47 ± 0.02 mMol DL-Alanine g^−1^ trypsin activity^−1^) were significantly higher compared to that of the SH and SS groups (0.14 ± 0.02 mMol DL-Alanine g^−1^ trypsin activity^−1^ and 0.22 ± 0.03 mMol DL-Alanine g^−1^ trypsin activity^−1^, respectively) (*p* < 0.05). Moreover, the SH group exhibited significantly lower digestibility than the SS group (*p* < 0.05) (Figure 4A). In terms of overall of *in vitro* protein digestibility in the experimental diets, we found that the LFM + TH diet group (0.75 ± 0.04 mMol DL-Alanine g^−1^ trypsin activity^−1^) showed a significantly higher value compared to all other diet groups (*p* < 0.05). However, no significant differences were observed between the HFM, LFM, and LFM + SS diet groups (0.63 ± 0.02 mMol DL-Alanine g^−1^ trypsin activity^−1^, 0.57 ± 0.02 mMol DL-Alanine g^−1^ trypsin activity^−1^, and 0.61 ± 0.03 mMol DL-Alanine g^−1^ trypsin activity^−1^, respectively), although these were significantly higher than in the LFM + SH diet groups (0.49 ± 0.01 mMol DL-Alanine g^−1^ trypsin activity^−1^) (*p* < 0.05) (Figure 4B).

#### 3.6.2. Apparent Digestibility Coefficients (ADCs)

The ADCs for dry matter, protein, and lipid are shown in Table 8. Fish fed the HFM, LFM + TH, and LFM + SS diets exhibited a significantly higher ADC of dry matter than fish fed the LFM and LFM + SH diets (*p* < 0.05). The ADC of protein was significantly higher in the fish fed with the LFM + TH diet compared to that of the LFM, LFM + SH, and LFM + SS diet groups (*p* < 0.05), but there was no significant difference compared to the HFM diet (*p* > 0.05). No significant difference was observed in the ADC of lipid among the treatments (*p* > 0.05).

### 3.7. Ammonia Stress Challenge Test

**Figure 5:**
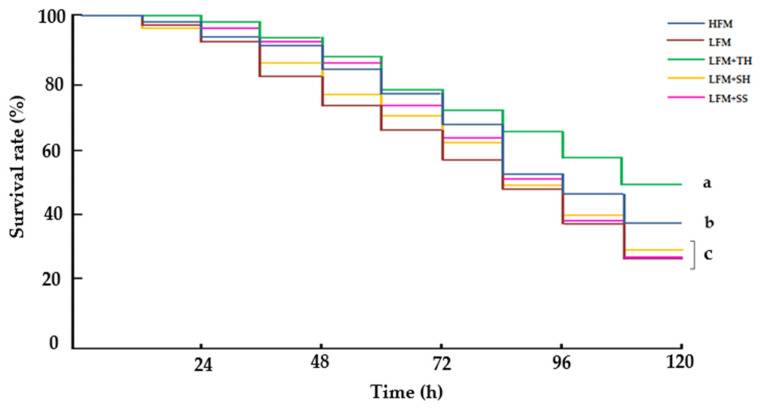
Survival rates of Asian seabass following a 120 h ammonia stress challenge (Kaplan–Meier survival method, followed by log-rank test, *p* < 0.05). Values are presented as means ± SD (*n* = 3). Different letters indicate statistical differences (*p* < 0.05) among the treatments. Abbreviations: HFM, high-fish-meal diet; LFM, low-fish-meal diet; LFM + TH, LFM diet + tuna hydrolysate; LFM + SH, LFM diet + shrimp hydrolysate; and LFM + SS, LFM diet + salmon silage.

After the ammonia challenge test, the survival of the fish fed on diets supplemented with marine hydrolysates range from 25.3% to 49.3% (Figure 5). The fish fed the LFM + TH diet showed the highest survival (49.3 ± 2.3%) compared to the other dietary treatments (*p* < 0.05). The HFM diet group (37.3 ± 2.3%) also had a significantly higher survival rate than the LFM diet group (*p* < 0.05). However, no significant differences were observed between the LFM (25.3 ± 2.3%), LFM + SH (29.3 ± 2.3%), and LFM + SS (25.3 ± 2.3%) groups (*p* < 0.05).

**Figure 6:**
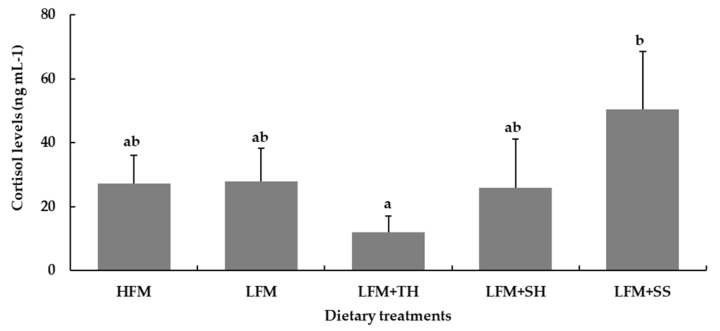
Cortisol levels in Asian seabass following a 120 h ammonia stress challenge. Values are presented as means ± SD (*n* = 5). Different letters indicate statistical differences (*p* < 0.05) among the treatments. Abbreviations: HFM, high-fish-meal diet; LFM, low-fish-meal diet; LFM + TH, LFM diet + tuna hydrolysate; LFM + SH, LFM diet + shrimp hydrolysate; and LFM + SS, LFM diet + salmon silage.

Cortisol levels in the blood plasma of Asian seabass tended to decrease in the fish fed with the LFM + TH diet, although no significant differences were observed compared to the HFM, LFM, and LFM + SH diet groups (*p* < 0.05). However, fish fed the LFM + SS diet exhibited significantly higher cortisol levels after 120 h of the ammonia challenge compared to all other dietary groups, though no significant differences were observed when compared to the LFM diet group (*p* < 0.05) (Figure 6).

## 4. Discussion

This study showed that supplementing marine hydrolysates, especially TH, in a LFM diet significantly improved growth and feed utilization in Asian seabass. The LFM + TH diet yielded the best results, fully restoring performance despite a 60% fish meal replacement that otherwise reduced growth by 19%. This improvement is likely due to enhanced palatability and nutrient balance, consistent with previous findings using 2.5–3% TH [7,16]. The superior performance of TH can be attributed to its favorable molecular weight distribution and digestibility characteristics. Specifically, TH contained a high proportion of low-molecular-weight peptides (<1000 Da, 54.1%) (Table 3), which are known to stimulate feed intake and improve nutrient absorption [7,8,16,38,39,40,41,42,43,44]. Additionally, the LFM + TH diet exhibited the highest *in vitro* protein digestibility (0.75 ± 0.04 mMol DL-Alanine g^−1^ trypsin activity^−1^) (Figure 4) and a significantly higher apparent protein digestibility coefficient (ADCp) compared to other treatments (Table 8), indicating a better bioavailability of nutrients. These factors collectively contributed to improved feed intake, growth rate, and feed efficiency in Asian seabass fed the TH-supplemented diet. Furthermore, it is hypothesized that amino acids and low-molecular-weight peptides in TH may activate taste receptor cells (TRCs), potentially stimulating orexigenic neuropeptides such as neuropeptide Y (NPY) and agouti-related protein (AgRP) [45,46,47,48,49,50,51], thereby enhancing appetite and feed intake. Once ingested, these peptides might also influence intracellular signaling pathways such as the target of rapamycin (TOR) and insulin-like growth factor (IGF-I), which are associated with protein synthesis and growth [40,51,52,53,54,55,56,57,58,59,60,61,62,63,64,65]. However, these proposed mechanisms require further experimental validation.

Hematological and immunological parameters are key indicators of fish health and nutrition [16]. In this study, marine protein hydrolysate supplementation did not significantly affect these parameters in Asian seabass, except for lysozyme activity. This aligns with previous studies in Asian seabass [66], pompano (*T. blochii*) [67], snakehead fish (*C. striata*) [8], and red seabream (*P. major*) [68,69], as well as olive flounder (*P. olivaceus*) [38,39,69], which also found no significant changes in hematological indices. However, several studies reported increased lysozyme activity with hydrolysate supplementation in Asian seabass [17], pompano (*T. blochii*) [67], olive flounder (*P. olivaceus*) [39], red seabream (*P. major*) [69], yellow croaker (*P. crocea*) [70], Japanese seabass (*L. japonicus*) [71], and juvenile coho salmon (*Oncorhynchus kisutch*) [72]. Some studies, however, found no effect on lysozyme activity [7,8,41,44,71]. Mechanistically, this may be attributed to bioactive peptides within the hydrolysates that resemble pathogen-associated molecular patterns (PAMPs), thereby activating pattern recognition receptors (PRRs) such as Toll-like and NOD-like receptors. This activation triggers downstream signaling pathways (e.g., NF-κB and MAPK), leading to an increased expression of immune-related genes, including lysozyme. Additionally, hydrolysates may modulate gut microbiota, reduce oxidative stress, and promote the cytokine-mediated recruitment of lysozyme-producing cells [73,74,75]. Overall, marine protein hydrolysates may not alter all hematological or immunological parameters but can influence non-specific immune responses like lysozyme activity. Effects may depend on species, hydrolysate concentration, and supplementation duration. Further research is needed to clarify these immune-modulating mechanisms and their health benefits.

Blood plasma metabolic markers parameters are commonly used as physiological indicators to assess the health status of fish [76]. In the current study, the dietary supplementation of marine protein hydrolysates had no significant effect on the blood plasma metabolic markers parameters of Asian seabass, which is consistent with previous studies [17]. Similarly, Pham et al. [67] found that dietary TH supplementation in pompano (*T. blochii*) had no significant impact on blood plasma metabolic markers parameters, except for total protein. On the other hand, fish fed diets containing 6.1% to 12.2% fish protein hydrolysates in Asian seabass showed significantly lower plasma glucose levels compared to the control group [66], a result also reported in olive flounder (*P. olivaceus*) and red seabream (*P. major*) fed LFM diets supplemented with TH or SH [69]. In contrast, Asian seabass fed LFM supplemented with 2.5% TH exhibited a significantly increased albumin level compared to those fed the LFM diet [7]. Although aspartate transaminase levels did not differ significantly among treatments, the LFM + SS group showed a notable reduction. This may reflect improved liver health or reduced physiological stress, as supported by previous findings on AST as a biomarker for hepatic function and stress response in Asian seabass [77].

Histological analysis showed normal liver architecture in all fish, with large hepatocytes arranged in cords and separated by sinusoids containing erythrocytes. This correlates with serum levels of creatinine, aspartate transaminase, and alkaline phosphatase, commonly used for the detection of liver damage or organ dysfunction in fish species [14,78,79].

The intestinal epithelium of fish, which includes villi, microvilli, and goblet cells, is highly responsive to dietary changes. A deficiency in nutrients and impaired absorption lead to structural alterations in the intestinal epithelium, such as a reduction in villi length and decrease in the number of goblet cells, ultimately compromising immune function [66]. In the present study, it was found that marine protein hydrolysates supplementation (TH, SH, and SS) significantly increased villi length and goblet cell count in the intestines of Asian seabass compared to the LFM diet, with TH-fed fish showing the most significant improvement. This increase in villi length and goblet cell count in TH-fed fish was even greater than in the HFM-fed fish group, indicating a full recovery of intestinal functions. Similar findings were reported in previous studies in various fish species, including Asian seabass [7,65,66], olive flounder (*P. olivaceus*) [38,39], red seabream (*P. major*) [38], and European seabass (*D. labrax*) [80,81]. The increase in villi length leads to a larger surface area in the intestine, improving nutrient absorption [82]. However, Domeneghini et al. [83] also revealed a positive relationship between higher goblet cell numbers and elevated mucosal membrane protection. Siddik et al. [65] also assumed that the increment of goblet cells in Asian seabass fed fish protein hydrolysates could be attributed to the enhanced immunity against invading microorganisms. Previous studies have not reported any histopathological changes in the livers of Asian seabass associated with soybean-meal-based diets [7,12]. While hepatic alterations such as cytoplasmic vacuolization and increased lipid accumulation were observed in juvenile Asian seabass fed diets containing more than 20% of fish protein hydrolysates, no such liver changes were observed in the present study. Additionally, biochemical parameters like creatinine, alkaline phosphatase, and aspartate transaminase did not show significant differences among the treatments. This suggests that the inclusion of marine protein hydrolysates in the diets did not cause any hepatocellular damage in Asian seabass.

Carcass composition results showed no significant differences in the whole-body proximate composition of Asian seabass supplemented with marine protein hydrolysates (TH, SH, and SS), consistent with prior studies [7,39,66,69] and indicating that these supplements improve growth and health but do not notably alter tissue nutrition.

*In vitro* assays showed that TH and fish meal (65% CP) had high protein digestibility (0.47 ± 0.02 mMol DL-Alanine g^−1^ trypsin activity^−1^), while the LFM + TH diet was highest at 0.75 ± 0.04. Other diets showed no significant differences. Enhanced digestibility is due to hydrolysis, which improves protein solubility and dispersibility and generates small peptides and free amino acids [18,84].

The protein ADC in the fish fed with the TH-supplemented diets was comparable to that of the HFM diet group, matching growth results. The LFM diet group showed lower digestibility, but TH supplementation restored it, consistent with findings in Asian seabass [66], olive flounder (*P. olivaceus*) [38,39,41,69], red seabream (*P. major*) [68], and Atlantic salmon (*S. salar* L.) [85]. This is due to enzymatic hydrolysis producing bioavailable peptides [65]. The SH and SS diets did not significantly improve ADC, likely due to excess free amino acids and low-MW peptides [65]. Lecduc et al. [80] found that TH or SH enhanced intestinal metabolism in European seabass (*D. labrax*). Improvements in gut morphology with TH or SH may have disrupted digestion, highlighting the need to optimize hydrolysate levels for balanced nutrient absorption.

Fish are known to be highly sensitive to stressors, often reacting to stimuli at levels undetectable by terrestrial animals. In this study, an ammonia stress challenge test revealed that fish fed the LFM + TH diet exhibited the highest survival rate (49.3 ± 2.3%) and significantly lower cortisol levels, indicating enhanced stress resistance compared to other dietary treatments. Although there is no direct evidence that tuna hydrolysate can suppress cortisol levels in Asian seabass or other fish species under ammonia-induced stress, it is hypothesized that bioactive peptides with antioxidant properties and amino acids functioning as neuromodulators present in tuna hydrolysate may contribute to cortisol regulation [15,80,86]. Notably, peptides such as Tyr-Glu-Asn-Gly-Gly, Glu-Gly-Tyr-Pro-Trp-Asn, Tyr-Ile-Val-Tyr-Pro-Gly, and Trp-Gly-Asp-Ala-Gly-Gly-Tyr-Tyr, previously isolated from tuna hydrolysate have demonstrated strong radical-scavenging activities and may play a role in modulating stress responses [86]. This effect is believed to occur through the downregulation of the hypothalamic–pituitary–interrenal (HPI) axis, leading to the inhibition of corticotropin-releasing hormone (CRF) secretion from the hypothalamus. Consequently, this suppresses the stimulation of corticotrophic cells in the anterior pituitary and reduces adrenocorticotropic hormone (ACTH) release, ultimately lowering cortisol synthesis and secretion by interrenal cells in the head kidney [87,88]. While data on stress and cortisol regulation in fish remain limited, improved tolerance to elevated ammonia levels may enhance survival during sudden environmental changes.

## 5. Conclusions

Dietary supplementation with 5% TH could effectively replace up to 60% of fish meal in low-fish-meal diets, with soybean meal compensating up to 42%. This supplementation significantly improved the growth, feed utilization, survival rate, diet digestibility, immune function, and intestine morphology in juvenile Asian seabass. Additionally, the TH diet enhances survival rates under ammonia stress, highlighting the potential of marine protein hydrolysate to improve stress tolerance. These findings suggest that marine protein hydrolysate supplementation offers a sustainable and efficient alternative to conventional fish-meal-based diets.

## Figures and Tables

**Table 1:** Chemical compositions, molecular weights, and amino acids profiles of tuna hydrolysate (TH), shrimp hydrolysate (SH), and salmon silage (SS).

Parameters	Ingredients		
	TH	SH	SS
Chemical composition (% dry matter)			
Dry matter	35.5	95.6	45.1
Crude protein	62.1	71.2	68.7
Crude lipid	8.2	9.2	19.1
Crude ash	20.2	12.3	6.7
Energy (MJ kg^−1^)	17.8	21.8	24.2
Soluble protein	>90	>90	n.d. *
Molecular weight (Dalton, % wet basis)			
<500	64	79	82
500–1000	5	10	7
1000–5000	15	10	9
5000–10,000	8	1	1
>10,000	8	0	0
Essential amino acids (% wet basis)			
Arginine	1.14	4.19	1.97
Histidine	0.85	1.03	0.46
Isoleucine	0.59	2.70	1.20
Leucine	1.09	4.04	2.31
Lysine	1.16	4.30	2.40
Methionine	0.40	1.30	0.95
Phenylalanine	0.59	2.90	1.17
Threonine	0.72	2.50	1.17
Tryptophan	0.15	0.80	0.37
Valine	0.77	3.20	1.51
Total essential amino acids	7.46	26.96	13.51
Non-essential amino acids (% wet basis)			
Alanine	1.34	4.30	1.85
Aspartic acid	1.37	5.70	2.86
Glutamic acid	2.12	9.00	4.31
Glycine	1.91	4.90	1.85
Proline	1.09	2.30	1.05
Serine	0.74	2.40	1.85
Tyrosine	0.47	2.30	0.28
Total essential amino acids	7.46	26.96	13.51
Total non-essential amino acids	9.04	30.90	14.05
Total amino acids	16.50	59.08	27.56

Abbreviations: TH, tuna hydrolysate; SH, shrimp hydrolysate; and SS, salmon silage. * n.d. = no data.

**Table 2:** Formulation and chemical composition of experimental diets for Asian seabass (% as-fed basis).

Ingredients (% Crude Basis)	Dietary Treatment				
	HFM	LFM	LFM + TH	LFM + SH	LFM + SS
Fish meal, 65%CP	25.00	10.00	10.00	10.00	10.00
Soybean meal, 44%CP	23.00	48.46	42.88	45.03	42.60
Poultry by-product meal	12.00	12.00	12.00	12.00	12.00
Wheat gluten	5.06	5.06	5.06	5.06	5.06
Wheat flour	28.29	14.13	15.01	15.56	15.69
Tuna crude oil	4.50	5.80	5.50	5.80	5.40
Choline chloride	1.00	1.00	1.00	1.00	1.00
Monocalcium phosphate	0.00	2.00	2.00	2.00	2.00
DL-Methionine	0.00	0.35	0.35	0.35	0.25
L-Lysine	0.10	0.20	0.20	0.20	0.00
Salt, NaCl	0.20	0.15	0.15	0.15	0.15
Vitamin premix ^1^	0.20	0.20	0.20	0.20	0.20
Mineral premix ^2^	0.15	0.15	0.15	0.15	0.15
Antioxidants ^3^	0.25	0.25	0.25	0.25	0.25
Antimicrobial agents ^4^	0.25	0.25	0.25	0.25	0.25
Tuna hydrolysate			5.00		
Shrimp hydrolysate				2.00	
Salmon silage					5.00
Analyzed chemical composition (% dry matter) ^5^					
Dry matter	92.50	89.52	90.69	89.60	89.39
Crude protein	48.84	48.51	48.43	48.09	48.18
Crude lipid	11.89	11.95	11.91	11.83	11.97
Ash	10.18	9.75	10.34	10.67	10.18
Crude fiber	2.34	2.52	1.64	1.62	2.61
Nitrogen-free extract	26.75	27.26	27.68	27.79	27.06
Gross energy (MJ kg^−1^)	19.92	19.62	19.70	19.61	19.36

Abbreviations: CP, crude protein; HFM, high-fish-meal diet; LFM, low-fish-meal diet; LFM + TH, low-fish-meal diet supplemented with tuna hydrolysate; LFM + SH, low-fish-meal diet supplemented with shrimp hydrolysate; and LFM + SS, low-fish-meal diet supplemented with salmon silage. ^1^ Vitamin premix (1 kg) = vitamin A, 3,000,000 mg kg^−1^; vitamin B1-Thiamin, 15,000 mg kg^−1^; vitamin B2-Riboflavin, 12,500 mg kg^−1^; vitamin B3-Niacin, 50,000 mg kg^−1^; vitamin B5-Pantothenic acid, 40,000 mg kg^−1^; vitamin B6-Pyridoxine, 20,000 mg kg^−1^; vitamin B7-Biotin, 750 mg kg^−1^; vitamin B9-Folic acid, 3000 mg kg^−1^; vitamin B12, 100 mg kg^−1^; vitamin D, 1,000,000 µg kg^−1^; vitamin E, 90,000 mg kg^−1^; and vitamin K, 20,000 mg kg^−1^. ^2^ Mineral premix (1 kg) = Ca, 150 mg kg^−1^; *p*, 150 mg kg^−1^; Cu, 20,000 mg kg^−1^; Fe, 40,000 mg kg^−1^; Mn, mg kg^−1^; Se, 280 mg kg^−1^; Zn, 40,000 mg kg^−1^; and I, 2200 mg kg^−1^. ^3^ Antioxidants = Butylated Hydroxytoluene (C_15_H_24_O). ^4^ Antimicrobial agents = Calcium propanoate (Ca(C_2_H_5_COO)_2_). ^5^ Analysis performed by the Agricultural Development Research Center in northeast Thailand, Faculty of Agriculture, Khon Kaen University: dry matter (AOAC official method 934.01), crude protein (Kjeldahl method, total N × 6.25), crude lipid (AOAC official method 2003.05, Soxhlet extraction), crude fiber (Fibertherm method), ash (AOAC official method 942.05), nitrogen-free extract (NFE) was calculated as % of dry matter—(% of crude protein + % of crude lipid + % of crude ash + % of crude fiber), and gross energy (automatic dynamic bomb calorimeter (IKA^®^ Werke, C5000, Staufen, Germany).

**Table 3:** Peptides profile and amino acids profile of experimental diets for Asian seabass.

Parameters	Dietary Treatments				
	HFM	LFM	LFM + TH	LFM + SH	LFM + SS
Peptides profile (Dalton, % wet basis) ^1^					
<500	48.8	49.1	50.7	40.4	49.1
500–1000	3.9	2.7	3.4	3.0	3.8
1000–5000	7.0	5.6	6.6	7.2	6.6
5000–10,000	3.9	4.0	4.1	5.1	3.8
10,000–20,000	9.1	9.4	5.6	11.2	9.8
>20,000	27.4	29.1	25.6	33.2	36.7
Essential amino acids (% dry matter) ^2^					
Arginine	2.60	2.70	2.48	2.72	2.52
Histidine	0.96	1.03	0.99	1.16	1.04
Isoleucine	1.70	1.67	1.57	1.64	1.68
Leucine	2.83	2.91	2.67	2.83	2.87
Lysine	2.65	2.72	2.59	2.81	2.73
Methionine	1.01	1.04	1.01	1.10	1.09
Phenylalanine	1.81	1.82	2.03	2.01	1.83
Threonine	1.26	1.33	1.22	1.33	1.32
Tryptophan	0.50	0.55	0.48	0.50	0.47
Valine	2.24	2.33	2.21	2.35	2.35
Non-essential amino acids (% dry matter) ^2^					
Alanine	1.80	1.79	1.65	2.03	2.05
Aspartic acid	3.39	3.56	3.20	3.53	3.16
Cystine + Cysteine	0.75	0.69	0.68	0.69	0.69
Glutamic acid	6.60	7.14	7.51	7.42	7.01
Glycine	2.24	2.19	2.14	2.29	2.31
Proline	2.39	2.35	2.45	2.68	2.59
Taurine	0.16	0.14	0.20	0.20	0.28
Tyrosine	1.36	1.38	1.50	1.66	1.55
Serine	1.77	1.98	1.73	1.94	1.83
Total essential amino acids	17.57	18.10	17.23	18.47	17.91
Total non-essential amino acids	20.48	21.22	21.06	22.44	21.47
Sum-total amino acids	38.05	39.32	38.29	40.90	39.38

Abbreviations: HFM, high-fish-meal diet; LFM, low-fish-meal diet; LFM + TH, low-fish-meal diet supplemented with tuna hydrolysate; LFM + SH, low-fish-meal diet supplemented with shrimp hydrolysate; and LFM + SS, low-fish-meal diet supplemented with salmon silage. ^1^ Molecular weight analyses were performed using size exclusion chromatography SEC-HPLC. ^2^ Amino acids profiles analyses were performed by SGS Thailand using the ISO13903, HPLC [24].

**Table 4:** Growth performance and feed utilization of Asian seabass fed the experimental diets for 8 weeks.

Parameters	Dietary Treatments					
	HFM	LFM	LFM + TH	LFM + SH	LFM + SS	*p*-Value
Initial body weight (g fish^−1^)	2.61 ± 0.01	2.62 ± 0.01	2.62 ± 0.01	2.62 ± 0.01	2.62 ± 0.01	0.067
Final body weight (g fish^−1^)	47.96 ± 0.83 ^a^	40.30 ± 1.27 ^b^	48.05 ± 0.94 ^a^	38.38 ± 1.48 ^c^	41.34 ± 0.35 ^b^	<0.001
Percent weight gain (%)	1736.3 ± 32.6 ^a^	1436.4 ± 47.2 ^b^	1737.1 ± 40.9 ^a^	1367.5 ± 52.7 ^c^	1476.2 ± 13.0 ^b^	<0.001
Average daily gain (g day^−1^)	0.81 ± 0.01 ^a^	0.67 ± 0.02 ^b^	0.81 ± 0.02 ^a^	0.64 ± 0.03 ^c^	0.69 ± 0.01 ^b^	<0.001
Specific growth rate (% day^−1^)	5.20 ± 0.03 ^a^	4.88 ± 0.06 ^b^	5.20 ± 0.04 ^a^	4.80 ± 0.06 ^c^	4.92 ± 0.01 ^b^	<0.001
Feed intake (g fish^−1^)	51.57 ± 2.04 ^a^	49.14 ± 2.07 ^b^	50.49 ± 0.98 ^a,b^	46.65 ± 1.98 ^c^	52.40 ± 1.05 ^a^	<0.001
Feed conversion ratio	1.16 ± 0.05 ^a^	1.42 ± 0.94 ^c^	1.14 ± 0.01 ^a^	1.34 ± 0.03 ^b^	1.38 ± 0.04 ^b,c^	<0.001
Protein efficiency ratio	1.80 ± 0.05 ^b^	1.58 ± 0.06 ^c,d^	1.86 ± 0.02 ^a^	1.59 ± 0.02 ^c^	1.53 ± 0.04 ^d^	<0.001
Nitrogen retention (%)	27.26 ± 1.07 ^a^	25.65 ± 1.06 ^b^	28.03 ± 0.53 ^a^	27.51 ± 1.11 ^a^	23.38 ± 0.46 ^c^	<0.001
Lipid retention (%)	33.71 ± 1.32 ^b^	30.50 ± 1.26 ^c^	35.29 ± 0.67 ^a^	29.81 ± 1.20 ^c^	30.55 ± 0.61 ^c^	<0.001
Intraperitoneal fat (%)	1.74 ± 0.39	1.48 ± 0.40	1.81 ± 0.33	1.81 ± 0.23	1.75 ± 0.29	0.505
Hepatosomatic index (%)	2.38 ± 0.26	1.95 ± 0.46	2.09 ± 0.34	2.43 ± 0.34	1.92 ± 0.34	0.090
Viscerosomatic index (%)	8.55 ± 0.62	8.41 ± 0.97	8.18 ± 0.42	8.75 ± 0.46	8.08 ± 1.00	0.615
Survival rate (%)	96.8 ± 3.0 ^a^	90.0 ± 2.7 ^b^	96.0 ± 2.4 ^a^	96.8 ± 3.0 ^a^	96.8 ± 2.3 ^a^	0.023

Note: Values are the means of five replicate groups and are presented as mean ± standard deviation. Values with different superscripts in the same row are significantly different (*p* < 0.05). Dietary treatments are abbreviated: HFM, high-fish-meal diet; LFM, low-fish-meal diet; LFM + TH, low-fish-meal diet supplemented with tuna hydrolysate; LFM + SH, low-fish-meal diet supplemented with shrimp hydrolysate; and LFM + SS, low-fish-meal diet supplemented with salmon silage.

**Table 5:** Hematological and non-specific immune response of Asian seabass fed the experimental diets for 8 weeks.

Parameters	Dietary Treatments					
	HFM	LFM	LFM + TH	LFM + SH	LFM + SS	*p*-Value
Red blood cells (×10^9^ cells mL^−1^)	1.72 ± 0.08	1.63 ± 0.15	1.66 ± 0.08	1.67 ± 0.16	1.64 ± 0.15	0.803
White blood cells (×10^7^ cells mL^−1^)	0.87 ± 0.16	0.88 ± 0.19	0.90 ± 0.20	0.83 ± 0.16	0.90 ± 0.12	0.946
Hematocrit (%)	39.5 ± 0.5	38.3 ± 1.68	38.8 ± 2.99	38.8 ± 2.41	38.1 ± 2.27	0.861
Hemoglobin (g dL^−1^)	11.73 ± 1.21	10.88 ± 1.14	11.22 ± 0.78	11.69 ± 0.68	10.62 ± 1.63	0.470
MCHC (g dL^−1^) ^1^	29.78 ± 3.01	28.61 ± 2.58	28.92 ± 1.37	29.95 ± 0.76	27.95 ± 3.55	0.691
NBT (absorbance 540 nm) ^2^	1.134 ± 0.290	1.087 ± 0.477	1.296 ± 0.367	1.103 ± 0.327	0.867 ± 0.452	0.555
SOD (%inhibition) ^3^	66.39 ± 4.48	59.29 ± 7.32	65.26 ± 4.82	53.56 ± 16.48	60.85 ± 13.78	0.350
CAT (unit mL^−1^) ^4^	33.46 ± 8.20	36.80 ± 10.32	34.01 ± 14.36	39.48 ± 10.04	45.43 ± 9.68	0.414

Note: Values are the means of five replicate groups and are presented as mean ± standard deviation. Dietary treatments are abbreviated: HFM, high-fish-meal diet; LFM, low-fish-meal diet; LFM + TH, low-fish-meal diet supplemented with tuna hydrolysate; LFM + SH, low-fish-meal diet supplemented with shrimp hydrolysate; and LFM + SS, low-fish-meal diet supplemented with salmon silage. ^1^ MCHC: Mean cell hemoglobin concentration. ^2^ NBT: Nitro blue tetrazolium activity. ^3^ SOD: Superoxide dismutase activity. ^4^ CAT: Catalase activity.

**Table 6:** Blood plasma metabolic markers of Asian seabass fed the experimental diets for 8 weeks.

Parameters	Dietary Treatments					
	HFM	LFM	LFM + TH	LFM + SH	LFM + SS	*p*-Value
Cholesterol (mg dL^−1^)	194.0 ± 11.7	194.9 ± 19.3	203.6 ± 24.3	201.5 ± 19.2	182.2 ± 12.0	0.390
Triglyceride (mg dL^−1^)	265.8 ± 55.5	230.9 ± 59.1	228.7 ± 27.1	203.0 ± 68.2	194.4 ± 46.2	0.274
Glucose (mg dL^−1^)	16.6 ± 7.3	23.7 ± 9.4	25.5 ± 14.9	18.9 ± 14.9	23.8 ± 9.0	0.714
Albumin (g dL^−1^)	1.41 ± 0.30	1.69 ± 0.20	1.70 ± 0.37	1.62 ± 0.50	1.73 ± 0.34	0.623
Total protein (g dL^−1^)	4.30 ± 0.32	4.42 ± 0.23	4.55 ± 0.41	4.67 ± 0.18	4.68 ± 0.34	0.157
Creatinine (mg dL^−1^)	0.26 ± 0.02	0.28 ± 0.03	0.27 ± 0.03	0.27 ± 0.04	0.26 ± 0.02	0.184
Aspartate transaminase (U L^−1^)	127.3 ± 52.2	127.3 ± 45.9	113.0 ± 35.7	122.0 ± 14.4	80.2 ± 12.1	0.873
Alkaline phosphatase (U L^−1^)	63.8 ± 6.7	60.0 ± 2.3	60.2 ± 12.5	59.0 ± 6.9	64.8 ± 6.8	0.701

Note: Values are the means of five replicate groups and are presented as mean ± standard deviation. Dietary treatments are abbreviated: HFM, high-fish-meal diet; LFM, low-fish-meal diet; LFM + TH, low-fish-meal diet supplemented with tuna hydrolysate; LFM + SH, low-fish-meal diet supplemented with shrimp hydrolysate; and LFM + SS, low-fish-meal diet supplemented with salmon silage.

**Table 7:** Carcass proximate composition (% dry matter) of Asian seabass fed experimental diets for 8 weeks.

Parameters	Dietary Treatments					
	HFM	LFM	LFM + TH	LFM + SH	LFM + SS	*p*-Value
Proximate composition (%) ^1^						
Dry matter	26.23 ± 0.09	26.53 ± 0.12	26.67 ± 1.20	26.00 ± 0.00	26.17 ± 0.38	0.759
Crude protein	48.29 ± 0.14	47.54 ± 0.08	48.28 ± 0.98	47.60 ± 0.09	47.33 ± 0.95	0.158
Crude lipid	12.00 ± 0.30	11.72 ± 0.43	12.05 ± 0.97	11.57 ± 0.13	11.85 ± 0.16	0.823
Crude Ash	12.56 ± 0.75	12.18 ± 0.08	12.69 ± 0.79	12.24 ± 0.00	12.61 ± 0.79	0.900

Note: Values are the means of five replicate groups and are presented as mean ± standard deviation. Dietary treatments are abbreviated: HFM, high-fish-meal diet; LFM, low-fish-meal diet; LFM + TH, low-fish-meal diet supplemented with tuna hydrolysate; LFM + SH, low-fish-meal diet supplemented with shrimp hydrolysate; and LFM + SS, low-fish-meal diet supplemented with salmon silage. ^1^ Analysis was performed by the Agricultural Development Research Center in northeast Thailand, Faculty of Agriculture, Khon Kaen University: dry matter (AOAC official method 934.01), crude protein (Kjeldahl method, total N × 6.25), crude lipid (AOAC official method 2003.05, Soxhlet extraction), and ash (AOAC official method 942.05).

**Table 8:** Apparent digestibility coefficients (%, ADC) of dry matter, protein, and lipid of the experimental diets for Asian seabass.

Parameters	Dietary Treatments					
	HFM	LFM	LFM + TH	LFM + SH	LFM + SS	*p*-Value
ADCd ^1^	73.1 ± 0.3 ^a^	68.8 ± 0.8 ^b^	73.5 ± 1.0 ^a^	69.4 ± 1.2 ^b^	70.4 ± 3.7 ^a,b^	0.035
ADCp ^2^	90.3 ± 0.6 ^a^	88.7 ± 0.3 ^b^	90.7 ± 0.6 ^a^	88.3 ± 0.1 ^b^	88.0 ± 1.1 ^b^	<0.001
ADCl ^3^	83.8 ± 1.5	79.6 ± 2.7	82.5 ± 2.9	78.1 ± 0.2	77.6 ± 3.9	0.066

Note: Values are the means of five replicate groups and are presented as mean ± standard deviation. Values with different superscripts in the same row are significantly different (*p* < 0.05). Dietary treatments are abbreviated: HFM, high-fish-meal diet; LFM, low-fish-meal diet; LFM + TH, low-fish-meal diet supplemented with tuna hydrolysate; LFM + SH, low-fish-meal diet supplemented with shrimp hydrolysate; and LFM + SS, low-fish-meal diet supplemented with salmon silage. ^1^ ADCd: Apparent digestibility coefficient of dry matter. ^2^ ADCp: Apparent digestibility coefficient of protein. ^3^ ADCl: Apparent digestibility coefficient of lipid.

## Data Availability

Additional data supporting the findings of this study are available from the corresponding authors upon reasonable request. Raw data and histological images used in this study can be accessed upon request.
